# In situ printing of liquid superlenses for subdiffraction-limited color imaging of nanobiostructures in nature

**DOI:** 10.1038/s41378-018-0040-3

**Published:** 2019-01-14

**Authors:** Boliang Jia, Feifei Wang, Hoyin Chan, Guanglie Zhang, Wen Jung Li

**Affiliations:** 1 Department of Mechanical Engineering, City University of Hong Kong, Hong Kong, Hong Kong S.A.R. China; 2 Shenzhen Academy of Robotics, Shenzhen, 518000 China; 3 Department of Chemistry, Stanford University, Stanford, CA 94305 USA

**Keywords:** Optics and photonics, Nanoscience and technology

## Abstract

The nanostructures and patterns that exist in nature have inspired researchers to develop revolutionary components for use in modern technologies and our daily lives. The nanoscale imaging of biological samples with sophisticated analytical tools, such as scanning electron microscopy (SEM) and transmission electron microscopy (TEM), has afforded a precise understanding of structures and has helped reveal the mechanisms contributing to the behaviors of the samples but has done so with the loss of photonic properties. Here, we present a new method for printing biocompatible “superlenses” directly on biological objects to observe subdiffraction-limited features under an optical microscope in color. We demonstrate the nanoscale imaging of butterfly wing scales with a super-resolution and larger field-of-view (FOV) than those of previous dielectric microsphere techniques. Our approach creates a fast and flexible path for the direct color observation of nanoscale biological features in the visible range and enables potential optical measurements at the subdiffraction-limited scale.

## Biophotonics: In situ printing of liquid superlenses for imaging nano-bio-structures

A method has been developed for printing biocompatible superlenses directly onto biological materials to allow the direct color observation of nanoscale features under an optical microscope. A superlens is a lens that uses special materials to go beyond the diffraction limit (a feature of conventional lenses that limits the fineness of resolution), and a team led by Boliang Jia and Wen Jung Li at the City University of Hong Kong devised a means of printing glycerol (a transparent liquid) superlenses directly onto butterfly wings. With that approach, the team was able to observe nanoscale features that had not previously been visible using a conventional optical microscope. The authors believe that with the future advent of advanced liquid superlenses, their fast, flexible method will allow the nanoscale structural inspection of both biological and non-biological samples.

## Introduction

The investigation of biological samples has entered the nanoscale regime with the emergence of biomimetics. Due to its diffraction limit, conventional optical microscopy can no longer satisfy the demands of this field. With the advancement of new analytical microscopy tools and computer-aided image reconstruction techniques, nanoscale-resolution imaging has been accomplished. For example, the color producing structures of insects (beetle, *Sericea sericea* and butterfly, *Morpho cypris*) were discovered more than 70 years ago via scanning electron microscope (SEM)^1^. This discovery provided a first glance below the diffraction limit at the detailed structures of some colored insects. At that time, all other optical methods could not be used to resolve such nanostructures. Since then, Morpho butterflies have become well known not only for their beautiful iridescent coloration but also, more importantly, for the distinct photonic properties of their wing scales^2^. The light-interfering properties resulting from their brilliant nanostructures have attracted great interest in the fields of nanophotonics and bioinspired materials research for decades^3,4^. The characteristic structures of butterfly wings have become a standard model for light manipulation^5–8^ and chemical sensing^9–11^. To study structures below the diffraction limit, high-resolution SEM and TEM techniques that require sophisticated sample preparation must still be used^5,12,13^. Furthermore, for studies on the coloration mechanisms and phylogeny of butterflies, SEM has been an indispensable tool^14–18^.

In previous studies, the physical structures of butterfly wings were comprehensively revealed using the high resolution of SEM. However, no study has demonstrated the direct optical observation of the subdiffraction-limited structure of wing scales. Approximately a decade ago, Wang et al.^19^ first developed a technique using silica microspheres placed on the top of sample surfaces to resolve subdiffraction-limited structures with a conventional optical microscope. Subsequently, Darafsheh et al.^20,21^ studied the crucial factors for super-resolution imaging and highlighted the importance of the working media^21,22^. The authors also demonstrated subdiffraction resolutions using high-refractive-index (*n*) microspheres such as barium titanate glass (BTG) in liquid-immersed conditions^20,23^. Since 2011, a variety of applications using microspheres have already shown significant resolution enhancements for confocal microscopes^24,25^ and fluorescence microscopes^26,27^. In addition, functional scanning components can be integrated with microspheres to achieve more precise manipulation of superlenses^23,28–30^. For example, our group demonstrated the capability of resolving 80-nm features under water-immersion conditions using 57-μm diameter BTG (*n* = 1.9) microspheres on an atomic force microscope (AFM) platform and achieved time-efficient large-area imaging with nanoscale resolution^30^. However, even without sophisticated tools and precise control of the distance between the microspheres and the sample surface, the achievable resolution could still be significantly reduced, especially for applications in liquid-immersed conditions^20,30,31^. The advantages of using high-refractive-index microspheres in aqueous media for the in vivo observation of biological samples have drawn great interest in recent years^26,27,30^. The applications that have been proposed are undoubtedly useful, especially for studying liquid-immersed samples, such as biological cells. However, for observing samples under dry conditions, i.e., in air, the high-refractive-index materials have limited resolutions due to the unfavorable refractive index ratio of the sphere and medium^19,20^. Recently, our team demonstrated similar super-resolution imaging capabilities when using BTG (*n* = 1.9) and polystyrene (PS, *n* = 1.59) microspheres in air^32^, even though the BTG images are “real” and PS images are “virtual”. In this paper, we present an in situ printed biocompatible glycerol superlens (SL) with a better resolution and larger field-of-view (FOV) than those of BTG microspheres under dry conditions. Glycerol is a transparent liquid with a relatively high refractive index (*n* = 1.47) capable of printing as droplets over a wide range of size. More importantly, glycerol has strong intermolecular interactions that makes it highly evaporation-resistant. While the evaporation rate for microsized water droplets is almost instant, in our work, by adding glycerol with a volume ratio of 50%, the printed droplets could exist for at least 1 day on a substrate without a significant change in size. We explored the printing of glycerol superlenses directly on a Morpho butterfly wing and revealed nanoscale features that had never been seen under a conventional optical microscope. Furthermore, we characterized the glycerol images with a standard central processing unit (CPU) integrated circuit (IC) sample and observed structures with sizes as small as 50 nm at a spacing of 200 nm.

## Results

### In situ printing of glycerol superlenses on butterfly wings

**Fig. 1:**
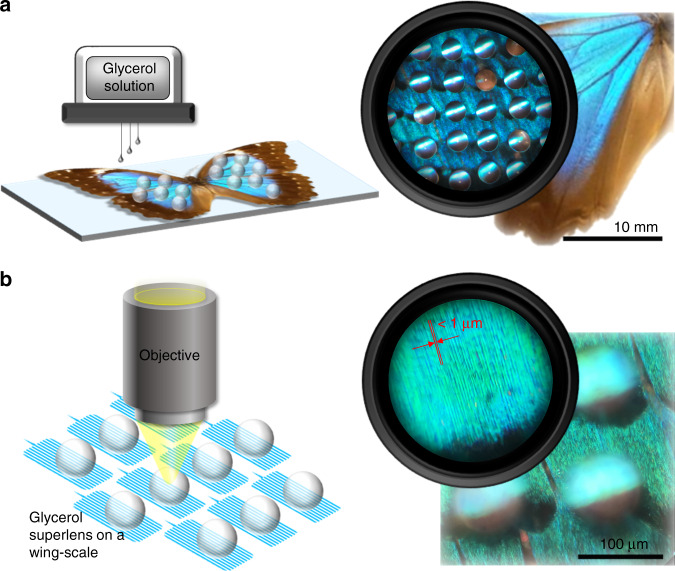
In situ printing of glycerol superlenses for the nanoscale imaging of butterfly wings. **a** Illustration of the printing process and a microscopic view of the formed superlens array on the wing scales. **b** Conceptual image of the direct nanoscale observation of butterfly wing scales via the superlenses, and the magnified image obtained through the superlens indicating a resolution of features with sizes less than 1 µm on the wing scale

The solid immersion lenses (SILs) enhance optical resolution by increasing the effective numerical aperture of the imaging medium^33^. The droplet lens can be considered as a liquid version of SILs having a flawless surface^34^. Therefore, the numerical aperture of our printed glycerol lens can theoretically be enhanced by a factor of *n* for hemispheres and *n*^2^ for superspheres^35^ under optimal conditions. The ink-jet printing machine was employed for dispensing the glycerol solution directly on the sample surface. The printing process is shown in Fig. 1. Butterfly wings are covered by arrays of wing scales with hydrophobic properties^36,37^. The surface “textures” of the scales also give rise to the hydrophobicity and allow the printed glycerol droplets to form transparent and close-to-complete microspheres. Through the microscope, the glycerol superlenses on top of the wing scales were clearly visible (Fig. 1a). By adjusting the focus, wing scale structures below 1 µm can be further magnified via the superlenses and some subdiffraction-limited features were able to be observed in color.

### Viscosity adjustment with respect to glycerol concentration

**Fig. 2:**
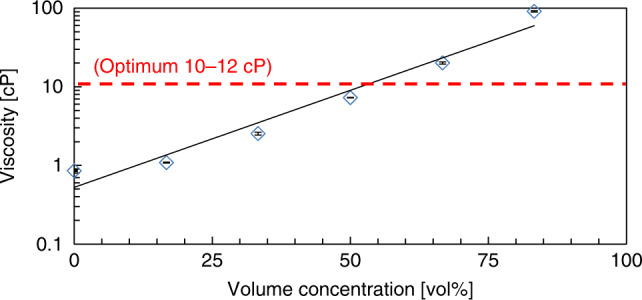
The change in glycerol solution viscosity with respect to volume concentration. The optimum range for printing is 10–12 cP according to the Fujifilm Dimatix Material Printer (DMP-2800) specifications. At least three measurements were recorded for each concentration

To obtain a jettable viscosity of the ink for the machine (2–30 cP, as suggested), a dilution test of the glycerol solution was performed. The glycerol solution was added into Milli-Q water to generate volume concentrations (vol%) of 0 (water only), 16.7, 33.3, 50, 66.7, and 83.3%. The viscosity was measured using a Brookfield DV2T viscometer (USA) at room temperature (22 °C). The average and standard deviations from at least three measurements are plotted in Fig. 2. The solution viscosity showed an exponential increase with volume concentration. Then, the 50 vol% solution (7.27 cP) was found to be the closest to the suggested viscosity for optimum performance (10–12 cP) and was used for the subsequent experiments. We successfully printed a solution with 67 vol% (20.17 cP) but failed to print a solution with 83.3 vol% (90.75 cP) due to the high viscosity.

**Fig. 3:**
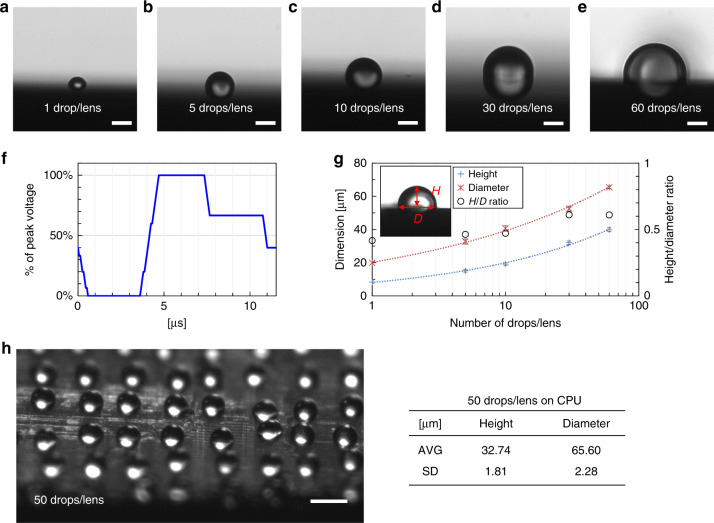
Characterization of printed glycerol superlenses with different numbers of drops/lens. **a**–**e** Lateral images of glycerol lenses with 1, 5, 10, 30, and 60 drops/lens on a clean silicon wafer. **f** The jetting waveform used in the experiment. **g** Plots of lens height (blue cross), diameter (orange star), and *H*/*D* ratio (black circle) with respect to the number of drops/lens. **h** An on-chip printed glycerol superlens array (50 vol%, 50 drops/lens) observed via a 4× (NA 0.10) objective at a 45° angle-of-view using a Nikon, Ti-E microscope (left). The table (right) shows the dimension statistics. Scale bar: **a**–**e** 20 µm, **h** 100 µm

By adjusting the jetting waveform, we could print high-quality droplets of the glycerol solution (50 vol%). We used a clean 76-mm (diameter) silicon wafer as a substrate and studied the dimensions of the printed glycerol superlenses formed from a different number of drops per lens. Figure 3a–e shows the lateral images of glycerol lenses with 1, 5, 10, 30, and 60 drops/lens. The images were taken using an inverted microscope (Nikon, Ti-E) with the wafer placed at a 90° angle to the objective which was achieved with a 360° rotatable fixture mounted on the sample stage of the microscope. The lateral images were acquired from the edge of the wafer. Figure 3f shows the jetting waveform of the printhead in operation, which is presented as a percentage of the peak voltage. In our experiment, 24 V and a 5 kHz jetting frequency (5000 drops/s) were used to print the 50 vol% glycerol solution. The average and standard deviations for the measured height (*H*) and diameter (*D*), as well as the *H*/*D* ratio, of the glycerol lenses are plotted in Fig. 3g with respect to the number of drops/lens; 20 samples were measured for each lens size. Both *H* and *D* showed a logarithmic increase with the number of drops/lens, while the *H*/*D* ratio stayed between 0.4 and 0.6, retaining a near-hemispherical shape for the superlens. One can also achieve different *H*/*D* ratios by modifying the hydrophobic characteristics of the substrate surface or by adding surfactants to the ink solution^38,39^. Moreover, the jetting frequency and printing area can be adjusted within the allowance of the equipment.

A standard CPU sample (22 nm lithography, Intel G2010) was used to characterize the imaging quality and to compare to that of the BTG microspheres. The initial diameter of the superlens was decided as approximately 60 μm, for which the BTG microspheres were commercially available. The number of drops/lens was chosen after a few experimental trials, and the resulting diameter of the glycerol lenses was comparable to that of the BTG microspheres. In our experiment, the CPU sample was decapsulated and cleaned thoroughly with ethanol. After drying, the sample exhibited similar hydrophobic characteristics to those of a silicon wafer. The 50 vol% glycerol solution was printed on the CPU surface using 50 drops/lens. Figure 3h shows a photo of the printed glycerol superlens array on the CPU sample from a 45° angle-of-view, which was achieved by tuning the rotation fixture to 45° from the objective. Then, 20 randomly selected on-chip printed lenses were measured, as described in the table on the right. The average diameter of the lenses was consistently approximately 65.60 μm with *H*/*D* ratios close to 0.5. Thus, the glycerol superlens was assumed to be an ideal hemispherical solid immersion lens (hSIL) in our study on this CPU sample.

### Imaging characteristics of the glycerol superlens

**Fig. 4:**
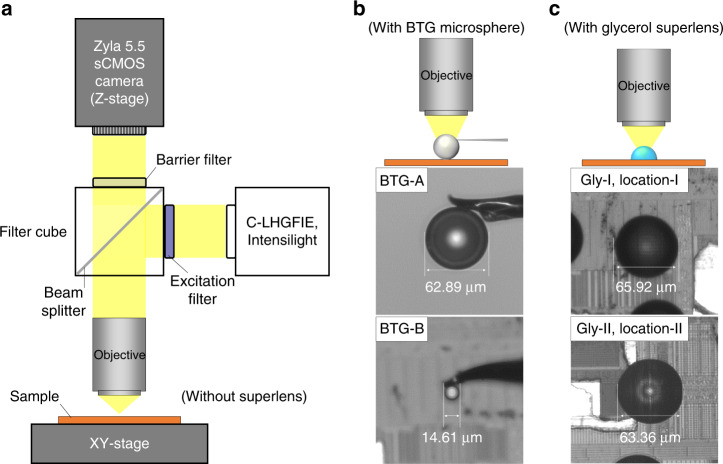
Configurations of the experimental setup. **a** Schematic of the imaging system based on the Nikon Ni-E platform without the use of a superlens. The major components include an Andor Zlya 5.5 sCOMS camera with a motorized focusing stage (Z), an Intensilight mercury-fiber illuminator (C-LHGFIE), a filter cube, an objective, and a motorized sample stage (XY). **b** The configuration with a BTG microsphere (top) and the optical images of two BTG microspheres, BTG-A (middle) and BTG-B (bottom), mounted on a microprobe (5 μm tip diameter) with NOA63 (Norland) adhesive. The diameter is 62.89 μm for BTG-A and 14.61 μm for BTG-B. **c** The configuration with a printed glycerol superlens (top) and the optical images of two lenses printed at location-I (middle) and location-II (bottom) of the CPU samples. The diameter is 65.92 μm for Gly-I and 63.36 μm for Gly-II. The microscope images in (**b**) and (**c**) are all top views with respect to the sample surface

Using the printed glycerol superlens arrays, we inspected several locations under a Nikon, Ni-E, the microscope system equipped with an Andor Zlya 5.5 sCOMS camera (5.5 megapixel) and motorized focusing stage (Z). The light source was an Intensilight mercury-fiber illuminator (C-LHGFIE). Other key components of the microscope include a switchable filter cube, objectives, and a motorized sample stage (XY). The 100× (NA 0.90) objective was used as the primary lens for the nanoscale imaging. The motorized stages and illumination were controlled by a PC with Nis-AR software. In this comparison experiment, all optical observations were acquired in air in reflection mode under white-light illumination. Figure 4 illustrates the configurations of the experimental setup. The conventional observation configuration is shown in Fig. 4a without the use of a superlens. When the BTG microsphere (Cospheric, 5–20-µm and 60-µm diameters, refractive index of 1.9) was to be used as a secondary lens, we glued the microsphere to a microprobe (PL-T5, Perfect LAB, with a tip diameter of 5 μm) and manipulated its position on a 3-axis micropositioner (PT300, Perfect LAB). The details of the microsphere and microprobe assembly were described in our previous work^40^. Figure 4b illustrates the BTG-in-air-configuration (top), and the assembled microspheres of BTG-A (middle) and BTG-B (bottom) having diameters of 62.89 and 14.61 μm, respectively. The smaller BTG-B was only used to obtain a relatively higher resolution than that achieved by BTG-A. Based on the comprehensive discussions and results from both simulations and experiments in refs. ^20,32^, we can conclude that, in general, large BTG microspheres could provide large FOVs and a wide range of focus, while a higher resolution could be achieved with smaller BTG microspheres. Figure 4c illustrates the configuration using a glycerol superlens (top) as a secondary lens and the two locations with printed lenses under inspection (middle, Gly-I at location-I; bottom, Gly-II at location-II). The diameters were 65.92 and 63.36 μm, respectively.

**Fig. 5:**
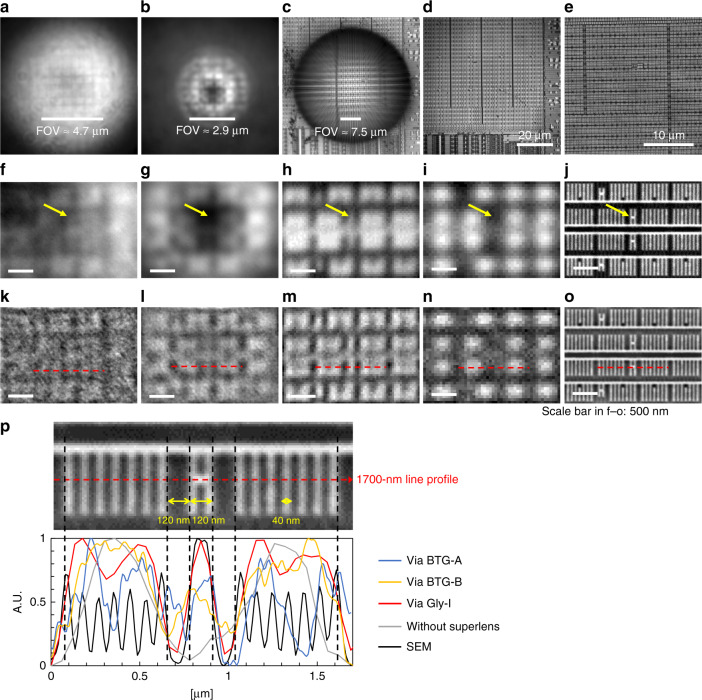
Experimentally acquired images at location-I on the CPU sample. **a**–**d** Optical images taken via BTG-A (**a**), BTG-B (**b**), Gly-I **c**, and without a superlens **d**. The objective used was 100× (NA 0.90). The estimated fields-of-view (FOVs) in **a**, **b**, and **c** are 4.7, 2.9, and 7.5 μm in diameter, respectively. **e** The SEM image over the same area. **f**–**j** Enlarged images over an approximate area of 3.9 μm × 2.7 μm from the center of **a**–**e**, respectively. The yellow arrows point to an “H”-like pattern approximately 120 nm in width. **k**–**o** Bandpass-filtered images of **f**–**j**, respectively. The scale bar in **f**–**o**: 500 nm. **p** Profiles of the red lines in **k**–**o** with normalized intensity. The 1700-nm line profiles are aligned with the features in the SEM image above

We compared the images taken from our rapidly fabricated glycerol superlens with those of BTG-A, BTG-B, and without a superlens. High-resolution SEM images were taken by FEG-SEM (FEI Quanta 450) as the reference for location-I and location-II under inspection. Figure 5a–d shows the optical images acquired in air at location-I via BTG-A, BTG-B, Gly-I, and without a superlens. Figure 5e shows the SEM image over the same area described above. The estimated FOV was approximately 4.7 μm in diameter for BTG-A and 2.9 μm for BTG-B, while Gly-I—with comparable size to that of BTG-A—had a significantly larger FOV of 7.5 μm. The corresponding magnification factors were 6.10× (BTG-A), 6.86× (BTG-B), and 1.34× (Gly-I). Figure 5f–j shows the approximately 3.9 μm × 2.7 μm areas enlarged from the center of the images in Fig. 5a–e, respectively. We can observe more nanoscale features in the superlens images (Fig. 5f–h) than in Fig. 5i, for example, the “H”-like pattern with an approximate width of 120 nm indicated by the yellow arrows. The ability to observe more nanoscale features is due to the limit of diffraction estimated using 0.61*λ*/*NA* (by the Rayleigh criterion). In our system, light below the 425-nm wavelength was cut off by the bright-field (BF) filter cube, and therefore, the best achievable resolution was no less than 288 nm. When comparing images obtained via glycerol (Fig. 5h) with those via BTG (Fig. 5f, g), the uniformity significantly improved, and the contours of the nanoscale features were sharper. Based on the SEM image, Fig. 5j, the characteristic widths of the patterns shown were 40 nm (bars) and 120 nm (“H” pattern). A line profile analysis was performed to compare image qualities using bandpass-filtered images (Fig. 5k–o; profile locations are indicated by red dashed lines). As shown in Fig. 5p, the 1700 nm profile covered a symmetrical pattern that had seven evenly distributed 40-nm-wide bars on both sides of the “H” pattern. The gaps between the bars were 40 and 120 nm on both sides of the “H” pattern. We applied a set of “size-matched” bandpass filters to images, Fig. 5f–j, to equally enhance the target features with sizes between 40 and 120 nm for comparison (supplementary materials Table S1). Among the treated images shown in Fig. 5k–o, we found that BTG-A (k) was still heavily distorted after bandpass filtering, while BTG-B (l) recovered more features, indicating a better resolution, which agreed with the previous reports^32^. All profiles shown in Fig. 5p were aligned based on the SEM profile for better representation. The Gly-I profile (red) matches more closely with the SEM profile (black) than with the profiles of BTG-A and BTG-B, especially for the 120 nm wide “H” pattern in the middle, indicating the superior resolution capability of the printed glycerol superlens compared with the resolution of the BTG microspheres, with both comparable and smaller sizes, in air.

### Filtered illumination

It is known that shorter illumination wavelengths can produce better image resolutions. We compared the longpass (for BF) filter and two other filters with center wavelengths at 480 and 520 nm for illumination, and the 480-nm filter could produce a higher resolution than that of the bright field. Via the glycerol superlens, features containing 50-nm-wide lines with 200-nm spacing were revealed under the 480-nm filter at location-II. The other two filters were unable to distinguish those features, and the results can be found in Figs. S1-S3 of supplementary materials.

### Observation of butterfly wing scales using glycerol superlenses

**Fig. 6:**
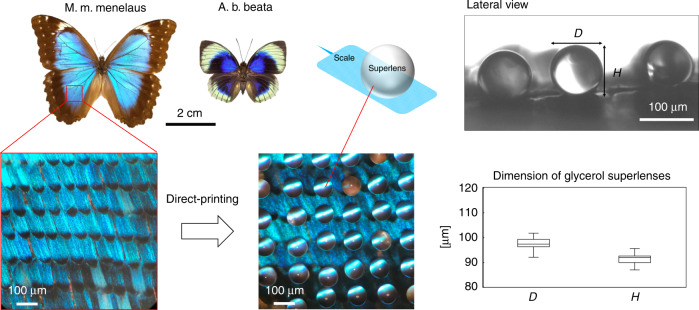
Schematic of the subdiffraction-limited imaging of a butterfly sample using in situ printed glycerol superlenses. The Morpho menelaus menelaus (M. m. menelaus) and Agrias beatifica beata (A. b. beata) samples were placed flat on a clean glass slide for printing. The microscopic images show the scale arrangement of the ventral wing of M. m. menelaus (bottom left) and the superlens array printed on the wing scales (middle). The superlenses exhibited a sphere-like geometry on the wing scales. The lateral image (upper right) was acquired using the inverted microscope (Nikon, Ti-E). The dimension statistics include data from 13 measured lenses based on their lateral images

In our work, we inspected two types of butterflies (Lepidoptera: Nymphalidae), namely, Morpho menelaus menelaus (M. m. menelaus) and Agrias beatifica beata (A. b. beata), both purchased from the Butterfly Company, Unltd., USA. Figure 6 shows photographs of the butterfly samples (product photos from the Butterfly Company website) and the dimensions of the printed superlenses. As observed from the microscopic images (bottom left and middle), the printed superlenses had uniform circular contours. From the lateral view image (upper right) and the dimension statistics (lower right), the height (*H*) and diameter (*D*) were very similar, indicating the sphere-like geometry of the superlenses, and can be clearly distinguished from those obtained on the CPU sample surface due to the differences in both material and the surface texture. The measured dimensions of the lenses on both kinds of butterflies were consistent. We printed 60 drops/lenses on both butterfly samples and obtained spherical glycerol lenses approximately 95 μm in diameter.

We used the upright microscope system (Nikon, Ni-E) to observe the wing scale features through the printed glycerol superlenses. The Morpho species have two types of wing scales, namely, ground and cover scales. For M. m. menelaus, the ground scales appear bright blue, and the cover scales appear translucent and lack pigment. The cover scales partially overlap with the ground scales^41^. The detailed anatomy of the wing scale can be found in the literature^2^, and a concise illustration is given in supplementary materials Fig. S4. Despite the intricate structures, our research was directed at the ridges and crossribs of the scales through the superlenses in this study. The ridges are longitudinal structures along the scale and are connected with crossribs in between. Each ridge consists of multiple layers of lamellae overlapping one another to form a shingle-like pattern.

**Fig. 7:**
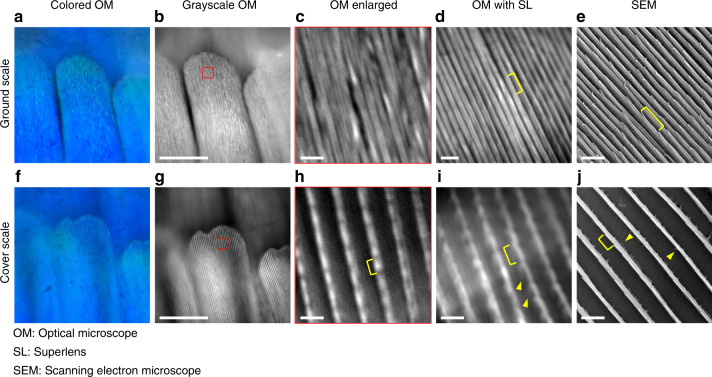
Comparison of images of the M. m. menelaus ventral wing scales. Color images **a** and **f** were taken from the eyepiece using an iPhone 7 Plus camera. Grayscale images **b**–**d** and **g**–**i** were taken with an Andor Zyla5.5 sCMOS camera. Images **e** and **j** were taken by SEM; **a**–**e** are images of ground scales; **f**–**j** are images of cover scales; and **c** and **h** are the enlarged images of the red square areas in **b** and **g**, respectively. Yellow brackets indicate one of the lamellae tips on the ridges. All optical images were taken under a 100× (NA 0.90) objective. Scale bar: 50 μm for **b** and **g**; 2 μm for **c**–**e** and **h**–**j**. The estimated extra magnifications produced by glycerol superlenses in **d** and **i** are 2.82× and 2.35×, respectively

However, these shingle patterns cannot be revealed under a conventional microscope. Figure 7 presents the captured images of the ground scales (a–e) and cover scales (f–j) of the M. m. menelaus sample. The images in Fig. 7a, b were taken under a 100× (NA 0.90) objective using color and monochromatic cameras, respectively. Figure 7c shows the enlarged region in the red square of Fig. 7b. The ridges, although having varying gaps at some locations, appeared to be merged with adjacent ridges. Figure 7d is the image taken through a glycerol superlens on another ground scale using the same objective. We observed a clear gap between two ridges and some “sections” along each ridge, one of which is marked with the yellow bracket. The SEM image in Fig. 7e was taken for comparison. As shown in Fig. 7e, the “section” was in fact the exposed portion or “tip” of the shingle-like lamella. The width of the lamella tip was approximately 100–200 nm. More accurate dimensions of the nanostructures can be obtained in a high-resolution SEM image of the ridge (supplementary materials Fig. S5a), in which the full width of each ridge was approximately 500 nm at 600-nm spacings, leaving an approximately 100–200-nm gap in between. Although the diffraction limit of the system is smaller than the full width of the ridges, their dense spacing caused a “merging” in the optical images of Fig. 7a–c. While these 100–200-nm gaps can be clearly distinguished with the superlens in Fig. 7d. However, the crossribs, being approximately 40–100 nm in width and 200 nm in length (between the adjacent ridges), were buried deep in between the adjacent ridges and were difficult to identify even with the high-resolution SEM unless the lamellae of the adjacent ridges were aligned perpendicular to the observation axis. Taking into account this fact, the glycerol superlenses were unable to observe the crossribs of the ground scales for this butterfly.

The same sequence of images applies for the cover scale in Fig. 7f–j. The cover scale has a substantially larger spacing between the ridges than does the ground scale. As a result, individual ridges can be distinguished even under a 10× (NA 0.30) objective (see supplementary materials Fig. S7). From the high-resolution SEM image of the cover scale ridge (supplementary materials Fig. S5b), we learned that the full width of each ridge was approximately 400 nm and that the spacing was approaching 2 μm. No crossribs exist on the cover scale. The tiny “roots” at the bottom of the ridges (called trabeculae^2^) are approximately 50 nm wide and less than 200 nm long. The lamellae tip sections can be barely identified in the conventional microscope image (Fig. 7h), while more “root-like” features can be observed through the glycerol superlens (Fig. 7i), as indicated by the yellow triangles. The images in Fig. 7c–e, h–j cover areas of approximately 10 μm × 10 μm. We assumed that the same types of scales possess consistent dimensions and structures over the inspected region.

**Fig. 8:**
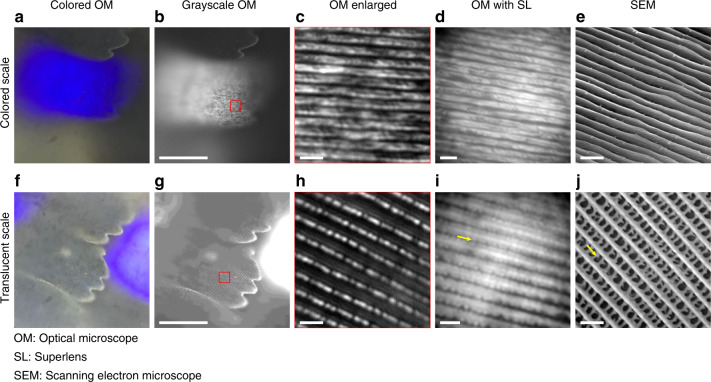
Comparison of images for the A. b. beata ventral wing scales. Color images **a** and **f** were taken from the eyepiece using an iPhone 7 Plus camera. Grayscale images **b**–**d** and **g**–**i** were taken by an Andor Zyla5.5 sCMOS camera; **e** and **j** were taken by SEM; **a**–**e** are images of ground scales; **f**–**j** are images of cover scales; and **c** and **h** are the enlarged images of the red square areas in **b** and **g**, respectively. Yellow arrows indicate the structures of crossribs between ridges. All optical images were taken under a 100× (NA 0.90) objective. Scale bar: 50 μm for **b** and **g**; 2 μm for **c**–**e** and **h**–**j**. The estimated extra magnifications produced by glycerol superlenses in (**d**) and (**i**) are 2.73× and 2.17×, respectively

In addition, we inspected another type of Nymphalidae butterfly, A. b. beata, which belongs to a different subfamily named Charaxinae. This butterfly was not as common as Morphinae in the biophotonics literature. As shown in Fig. 6, the bluish colors on the A. b. beata’s wings are darker than those on the wings of M. m. menelaus. Therefore, we studied the dark-blue regions of the wing. A. b. beata also has two types of scales. These scale types have not been previously clearly defined; therefore, we refer to them as “colored scales” and “translucent scales”. Figure 8 shows a comparison of the acquired images of these two types of wing scales following the same sequence shown in Fig. 7 (a–e for colored scale and f–j for translucent scale). From the enlarged area of the 100× image in Fig. 8c, we observed coarse ridges with clear gaps. The glycerol image (Fig. 8d) did not reveal many new features, but it was sharper than Fig. 8c. The SEM image (Fig. 8e) revealed a tilted array of ridges and showed that the lower lamellae were wider than their tips and could occupy the full width of the ridge up to 600 nm. Based on the high-resolution SEM image taken from the vertical direction (supplementary materials Fig. S6a), the gaps between ridges could reach 300 nm, which were above the diffraction limit of the optical system; however, the lamellae tips on the ridges were approximately 100 nm, which are narrower than those found on the ground scales of M. m. menelaus. As a result, the lamellae tip sections were harder to distinguish, and the underneath width-increasing lamellae layers (with approximately 60-nm separations) could be observed from the top. Furthermore, all underneath lamellae have an oblique growth at a small angle with respect to the scale substrate^41^, we suspect these could be the reasons why the ridges appeared coarse and “twisted” in the optical images (in Fig. 8c, d).

The 100× image (Fig. 8h) of the translucent scale of A. b. beata shows only stripe-like ridges and numerous “break points” along them, while the glycerol image (Fig. 8i) reveals additional patterns in between the ridges. The SEM image (Fig. 8j) indicates that the patterns seen in Fig. 8i were due to the presence of crossrib networks, approximately 200 nm in width. The glycerol superlenses, although unable to resolve the complete structure, proved the existence of “substructures” between the ridges. These comparisons demonstrate that in situ printed glycerol superlenses can extend the limit for observing nanoscale structures in biological samples.

### Colored image of subdiffraction-limited structures

**Fig. 9:**
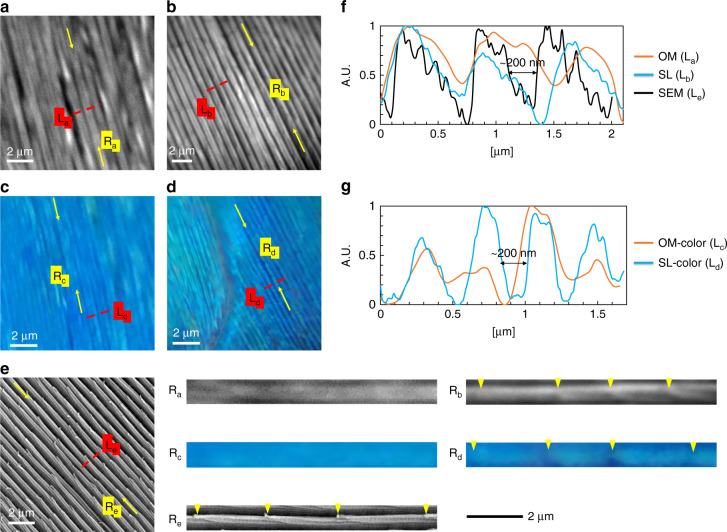
Analysis with color images of sub-diffraction-limited structures. Ground scales of M. m. menelaus was used for this analysis. **a**, **b**, and **e** are the same as in Fig. 7c, d, and e, respectively. **c** and **d** were taken from the eyepiece using an iPhone 7 Plus camera without and via the glycerol superlens. Line profiles over the red dashed lines in **a**–**e** are shown in **f** and **g**. The ridges marked by yellow arrows were enlarged and are shown in the lower right. The inverted yellow rectangles mark the identified lamella tips along each enlarged section of the ridges. For ridges R_a_ and R_c_ imaged without the superlens, no lamella tips could be distinguished. The labels “L_a–e_” correspond to line profiles, and labels “R_a–e_” correspond to the enlarged ridges. Scale bar: 2 μm. OM optical microscopy, SL superlens, SEM scanning electron microscopy

Based on the above observation, we found that the gaps between ridges on a ground scale of M. m. menelaus were approximately 200 nm and could be clearly revealed via a printed glycerol superlens. We performed further analysis of this structure with additional colored images, as shown in Fig. 9. The same images in Fig. 7c–e were reused as Fig. 9a, b, and e, respectively; Fig. 9c, d are color images of similar ground scales without and via the glycerol superlens. As mentioned previously, we could clearly identify the gaps between ridges and lamella tips along the ridges in the superlens images (Fig. 9b, d). We performed line profile analyses for all images (Fig. 9a–e), and the corresponding locations are marked as “L_a–e_”. We compared the line profiles for grayscale and colored images separately (Fig. 9f, g), as the images were taken using different cameras and optical configurations. The magnification factor by the superlens (Fig. 9d) is ~3.10 relative to Fig. 9c, and clear valleys of 200 nm could be seen in L_d_. Compared to the grayscale profile L_b_ in Fig. 9f, the colored profile was sharper. In addition, one of the ridges was extracted from each image (a–e) and enlarged in the lower right corner of Fig. 9 (“R_a–e_”). The identified lamella tips are marked by inverted yellow triangles. For both grayscale and colored images without using superlens, we could not identify any clear lamella tips along ridges R_a_ and R_c_. This experiment shows the capability of color-imaging sub-diffraction-limited nanobiostructures using glycerol superlenses.

## Discussion

We demonstrated a rapid fabrication scheme to print glycerol superlenses directly on both biological and nonbiological samples (for nonaqueous environments) and observed nanoscale structures. The evaporation-resistant microdroplets work as liquid-form micro-SILs on the sample surface. The shape of the liquid lens is dependent on the material hydrophobicity and micro-to-nanoscale textures of the surface. We demonstrated that glycerol droplets could be formed into hemispheres on the silicon-based IC surface of a CPU and near-spherical lenses on the more hydrophobic surface of butterfly wings. The refractive index of the glycerol superlens can be adjusted by the concentration, for example, an index of refraction of approximately 1.4 can be expected using a 50 vol% glycerol–water solution. Compared to conventional applications of SILs, one apparent advantage is that these liquid lenses can provide a better contact between the sample surface and the lens’ surface, while SILs may require index-matching liquids in between. In addition, the technique proposed in this paper enables the fast fabrication of microscale lenses (commercial SILs are typically of millimeter-scale), and hence, the printed glycerol lenses could allow the focus of source light spots into the optical wavelength scale, thereby enhancing the image solving capability. Moreover, although fabrication methods for microscale SILs exist, such as thermal reflow, laser writing, and mask photolithography, they are typically significantly more complicated and costly, not to mention the difficulties in avoiding structural defects during these fabrication processes. In contrast, the liquid lens’ surface is flawless, and its size can be simply controlled by drop-on-demand printing techniques. Last but not least, the printing of liquid lenses can utilize a wide range of liquid properties including refractive indices and surface tensions for various applications. To optimize the geometry of the printed superlenses, suitable conditioners or surfactants can be added to the glycerol-based solution subject to different substrate properties. Meanwhile, the viscosity of the solution is critical for drop-on-demand dispensing systems; therefore, the mechanical and electrical properties of the liquid used to create the droplet-lens have to be carefully considered.

We compared the imaging capability (with air as the medium) of the printed glycerol superlens with that of BTG microspheres that are comparable to those of our previously reported work^22^. We note here that many research efforts have been carried out over the past decade in the field of microsphere-based super-resolution imaging, including using relatively low refractive indices, small-sized spheres such as silica (*n*~1.46, diameter 2–9 μm) in air^19^, and using relatively high-refractive-index spheres such as BTG (*n*~1.9–2.1) under liquid-immersion conditions^20,23^. The refractive index ratio between the microsphere and its surrounding medium is one of the key factors in achieving the highest resolution for microspheres. However, the liquid-immersion scheme is not suitable for our purpose in this study. For air-medium observations, we can expect to obtain more comparable data to that of the glycerol superlens using a low-index microsphere (1.4–1.47) with a calibrated standard sample.

The positioning of microspheres for super-resolution microscopy has been an important issue^30,42,43^. In our previous work^40^, the assembled BTG microsphere with a metallic microprobe was manipulated by a micropositioner. When descending the microsphere towards the IC surface, we observed a slight dislocation with respect to the probe tip upon contact. In addition, we observed “Newton Rings” radiating from the center of BTG microspheres, as is commonly observed when there is contact between a sphere and a flat surface. The same principle applies for BTG in this work. On the other hand, the positioning of droplets in this work was controlled by the 3-axes moving stage of the commercial liquid dispenser. The mechanical repeatability is ±25 μm according to the system’s specifications. However, taking into account the variations in cartridge (the liquid holder) mounting and sample flatness (i.e., IC and butterfly wings), we can roughly control the position offset of the first droplet to within 100 μm with respect to the designated printing spot in experiments, which does not provide a precise spatial control of the dispensed lens compared to that of the aforementioned works. Nevertheless, the position of the liquid lens is not a critical issue in this work. To improve the accuracy in the positioning of the printed droplets, it is possible to custom-build a dispensing system with a 3-axes moving stage of significantly more accurate position resolution.

Compared with that of air-immersed BTG microspheres, our printed glycerol superlenses could offer a substantially larger FOV (7.4–7.5 μm in diameter) and significantly better resolved images. For solid-state samples, such as ICs, cleaning and repeated printing are feasible without damaging the sample due to the good solubility of glycerol in water and could be a simple and flexible application for IC failure analysis.

Two butterfly samples were investigated using in situ printed glycerol superlenses. We revealed nanoscale features of the wing scales that have never been resolved by conventional microscopes before. The narrow gaps of approximately 200 nm between ridges on the ground scales of M. m. menelaus were clearly resolved in colored images, whereas the ridges merged together when observed without the superlens. We noted that the color images of butterfly wings sometimes have better contrast than those taken with monochromatic cameras. This may be due to the color contrast effect of the sample, especially those having special optical characteristics like that of Morpho butterflies. This highlights the importance of “real-color” super-resolution inspection in the future. The additional features observed with glycerol superlenses, but not with the 100× objective solely, were the lamella tips on the ground scale ridges and trabeculae at the bottom of the cover scale ridges for M. m. menelaus and crossrib networks on the translucent scales for A. b. beata. Although in this experiment these features were not fully revealed compared to those in the SEM images, our approach demonstrates a simple preparation process and cost-effective high-resolution imaging.

Additionally, some water-immiscible liquids having high refractive indices may also be used for printing liquid lenses that may be used for water-immersion imaging applications. For example, the organic liquid diiodomethane (CH_2_I_2_) (*n*~1.77) can increase its refractive index to 1.80^44^ with dissolved antimony tribromide salts, although such compositions exhibit a high toxicity. More biocompatible liquids, such as silicone oil (a refractive index typically between 1.5 and 1.7) or other polymers, could be explored for superlens applications underwater using ink-jet printing or other dispensing methods. Finally, with advanced liquid superlenses being developed in the future, this scheme may become a fast and easy-to-implement solution for the nanoscale structural inspection of both biological and nonbiological samples that will inspire more interesting ideas in biophotonics.

## Materials and methods

### Butterfly samples

Dried Morpho menelaus menelaus (M. m. menelaus) and Agrias beatifica beata (A. b. beata) butterflies were purchased from the Butterfly Company Unlimited (formerly Butterflies and Things, IL, USA) and were used as received for in situ printing and the observation of nanostructures. The butterfly samples were mounted with a flat orientation on a clean glass slide suitable for use in a drop-on-demand ink-jet printing machine (Fujifilm Dimatix Material Printer, DMP-2800, Japan). The glycerol (≥99.5%, Sigma) was diluted to obtain a printable viscosity.

### Image processing and analysis

The image processing and analysis were conducted using ImageJ software (Fiji). The SEM and 100× optical images (without superlenses) had calibrated pixel sizes of 0.0135 nm/px (for Fig. 5) and 0.065 μm/px, respectively. As a result, the SEM image had a magnification factor of 4.34× compared to the 100× image pixel size. Similarly, by comparing the pixel numbers over the 1700-nm profile, we can estimate the pixel size of BTG-A, BTG-B, and Gly-I images. The “image-wise” magnification factors are inversely proportional to the pixel numbers. For instance, from the SEM image, we know the true features are sized 40 and 120 nm in size, and then, the filter size is set 2–10 px, which will suppress all features below 27 nm and larger than 135 nm as a result. An equivalent treatment was applied to other types of images with “size-matched” bandpass filters. The detailed parameters are listed in Table S1 in supplementary materials.

### Scanning electron microscopy characterization

Field-emission gun scanning electron microscopy (Model: Quanta^TM^ 450 FEG-SEM, FEI) was used for the SEM imaging of the CPU and butterfly samples. The beam voltage was 20 keV with a spot size of 4.0 for high-resolution imaging. Prior to the inspection, the butterfly samples were deposited with a thin layer of Au by a sputtering coater (Model: Q150TS Dual target sputtering system, QUORUM).

## Supplementary Information

Supplemental materials
