# Supplementary material for: In situ printing of liquid superlenses for subdiffraction-limited color imaging of nanobiostructures in nature
Source: Microsyst Nanoeng. 2019 Jan 14;5:1. doi: 10.1038/s41378-018-0040-3 (PMC6330505; doi:10.1038/s41378-018-0040-3)
Supplement: Supplementary file 1 — Supplemental materials [file 41378_2018_40_MOESM1_ESM.docx]

Title

In situ Printing of Liquid Superlenses for Sub-diffraction-limited Color Imaging of Nano-bio-structures in Nature

Authors:

Boliang Jia^1^, Feifei Wang^2,3^, Hoyin Chan^1*^, Guanglie Zhang^2^, and Wen Jung Li^1,2#^

1. Department of Mechanical Engineering, City University of Hong Kong

2. Shenzhen Academy of Robotics, Shenzhen, 518000, China

3. Department of Chemistry, Stanford University, Stanford, CA 94305, USA.

^*,#^Contact Authors: hoychan@cityu.edu.hk and wenjli@cityu.edu.hk

Supplementary Materials

**Filtered illumination**

The test schematic is shown in Fig. S1. In this experiment, Filter-1 is the bright-field condition that all wavelengths could pass though as used in previous experiments. Filter-2 is the excitation filter from a FITC cube (Nikon) having a centered wavelength at 480 nm with a 30 nm bandwidth. Filter-3 is the excitation filter from a Cy5 cube (Nikon), having a centered wavelength at 620 nm and a bandwidth of 60 nm.

We inspected location-II on the CPU sample with a printed glycerol superlens (Gly-II, 63.36 μm in diameter) under Filters-1, -2 and -3. Fig. S2a shows an optical image of Gly-II under a 100x (NA 0.90) objective. Its FOV (white dashed circle) has a diameter of approximately 7.4 μm. The yellow rectangle (approximately 3.6 μm x 2.9 μm) includes one of the repeating “blocks” at the center of the FOV. Fig. S2b-d on the right are the enlarged images of the unit block taken with Filter-1 (bright-field), Filter-2 (480 nm (30 nm)), and Filter-3 (620 nm (60 nm)), respectively. Fig. S2e shows the corresponding SEM image. We found that the Filter-2 image (Fig. S2c) revealed more details than the other two images. The red dashed lines in Fig. S2b-e covered our features of interest, which could not be seen clearly in Fig. S2b and d. Then, we plotted the line profiles with a normalized intensity in Fig. S2f before applying any additional processes to the images. The profiles were aligned with an enlarged but transposed SEM image showing the features of interest being three 50-nm wide lines with 200-nm gaps between. Filter-2’s profile (blue) clearly shows three peaks corresponding to three 50-nm wide lines, while the other two Gly-II profiles could not, indicating that the resolution of a glycerol superlens can be further enhanced by using a shorter wavelength with a narrow bandwidth.


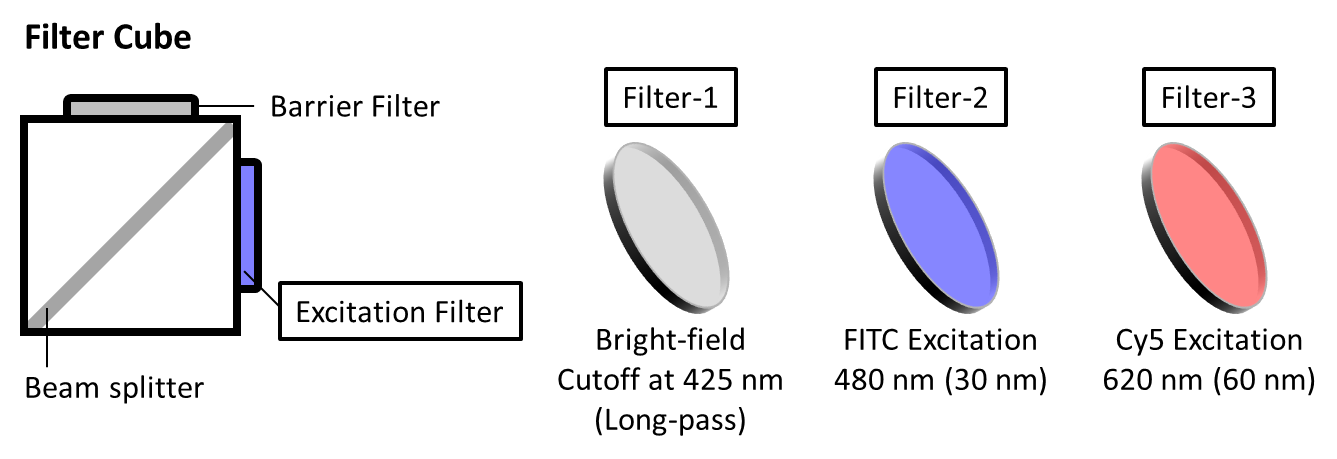


**Figure S1. Filter cube setup for wavelength test.** Filter-1: bright-field filter is a long pass filter with cutoff wavelengths at 425 nm, and all longer wavelength can pass. Filter-2: center wavelength at 480 nm with a 30 nm bandwidth (excitation filter from a FITC filter cube, Nikon). Filter-3: center wavelength at 620 nm with a 60 nm bandwidth (excitation filter from a Cy5 filter cube, Nikon). The filter cube was adapted from the bright-field filter cube, having a beam splitter and an empty Barrier Filter.


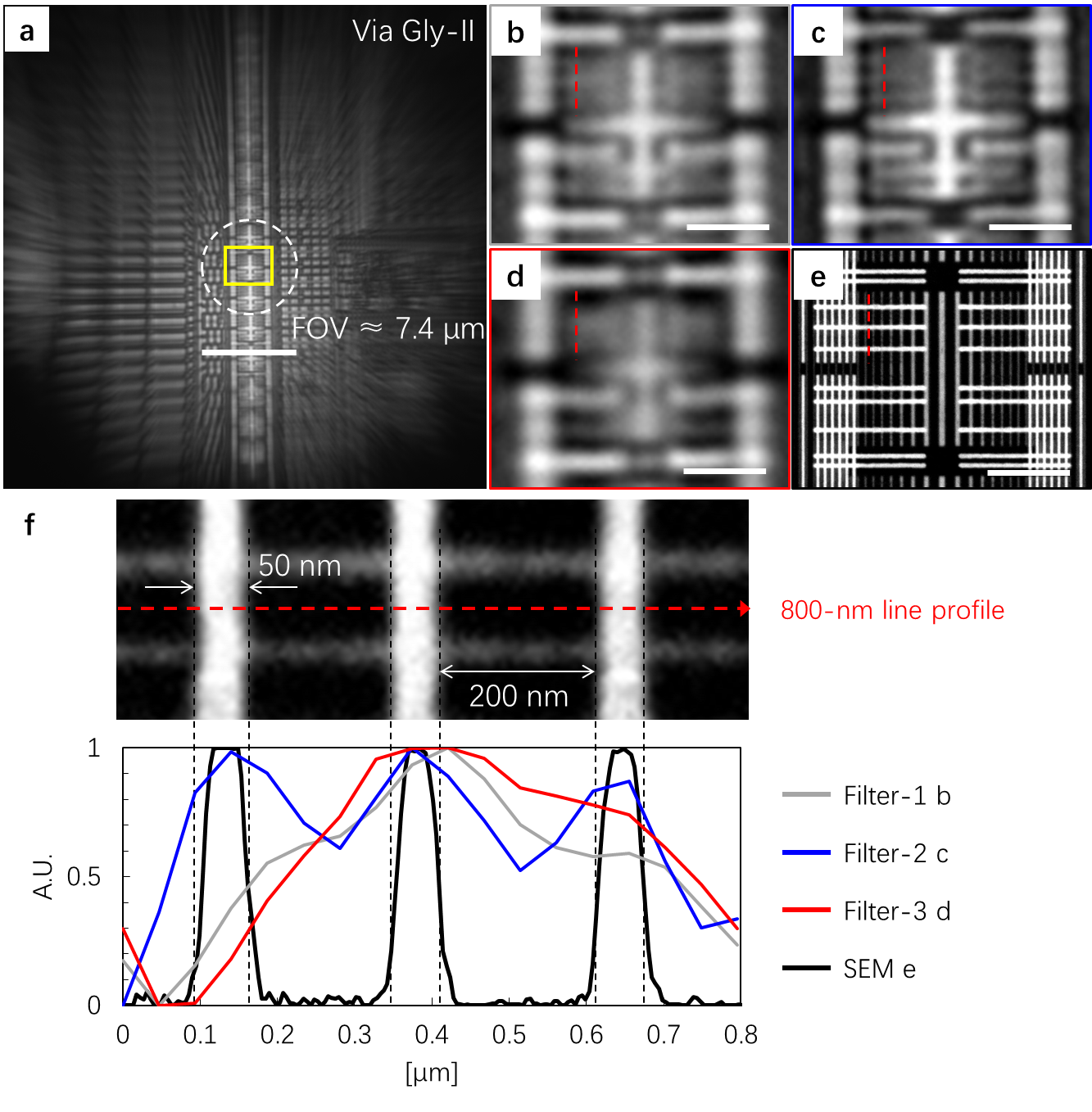


**Figure S2. Wavelength test using a glycerol superlens at location-II. a** Optical image of Gly-II (63.36 μm in diameter). The estimated FOV of 7.4 μm in diameter is indicated by the white dashed circle. The yellow rectangle shows an approximate area of 3.6 μm x 2.9 μm containing the features of interest. **b-d** The yellow rectangle area in **a** taken using Filter-1 (bright-field, all-pass), Filter-2 (centered at 480 nm with band width of 30 nm), and Filter-3 (centered at 620 nm with band width of 60 nm), respectively. **e** SEM image of the same area. Scale bar: 1 µm. **f** Profiles with normalized intensity over the red lines in **b-e**. Bandpass filtering was not applied in this analysis. In this experiment, the same light source and 100x (NA 0.90) objective were used.

As demonstrated earlier, BTG with a comparable size (BTG-A, 62.89 μm in diameter) was already unable to reveal 120 nm features clearly, thus it was less suitable for observing smaller features. Therefore, only the BTG-B image using a Filter-2 was shown for comparison. We focused on the same area and location as in Fig. S2 for the line profile analysis. Fig. S3a-d shows images after bandpass filter treatment for BTG-B, Gly-II, without a superlens, and SEM, respectively. The profiles from the red dashed lines in Fig. S3a-d are plotted in Fig. S3e with a normalized intensity. The same method was used to determine the “size-matched” bandpass filter for each image. The details of the magnification factors and filter size are listed in Table-S2 in the supplementary materials. We noticed that even after bandpass filtering, the profile from BTG-B was still highly distorted and unable to recover the three 50-nm wide lines from the profile, which is proof of the superior resolving power of the glycerol superlenses over the BTG microspheres for nanoscale imaging. Additionally, the FOV for BTG-B was approximately three times smaller than that of Gly-II in this experiment.


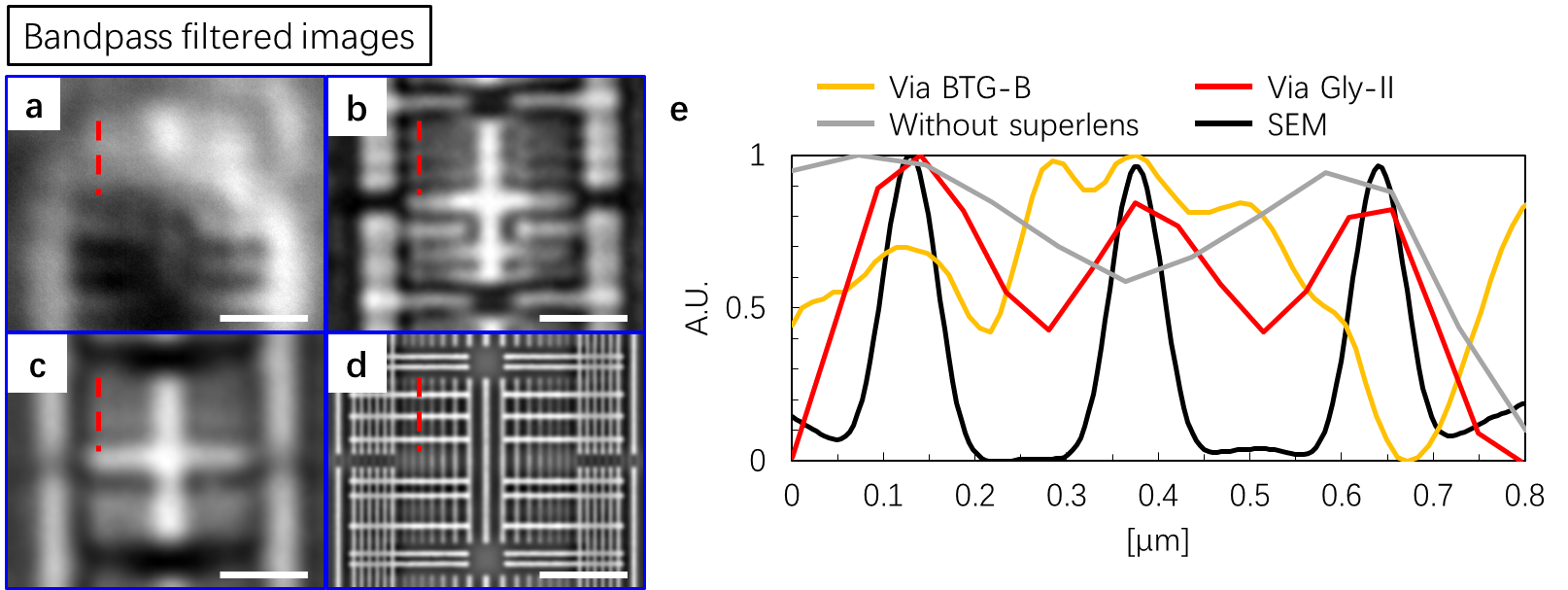


**Figure S3. Comparison of BTG-B, Gly-II and without a superlens using Filter-2.** **a** BTG-B. **b** Gly-II. **c** Without a superlens. **d** SEM image. Red dashed lines are 800 nm long and are at the same location as in Fig. S2. Scale bar: 1 µm. **e** Plot of the line profiles from **a-d** with normalized intensity. All images were processed with “size-matched” bandpass filters in the same manner as described earlier.

Butterfly wing scales


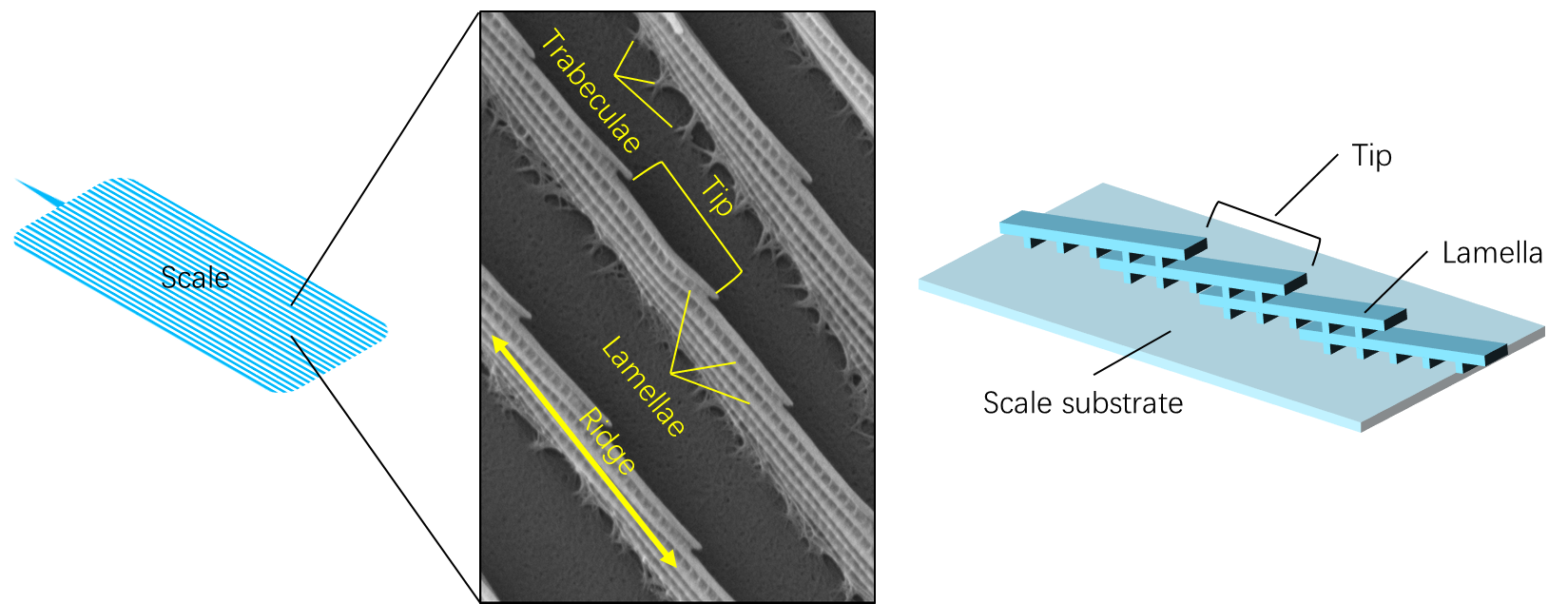


**Figure S4. Model of a butterfly wing scale structure.** The longitudinal structure along the scale is called a “ridge”. Each ridge is composed of multiple layers of lamellae overlapping one after another, leaving a section of the tip lamellae exposed. The root structures called trabeculae connect ridges to the substrate.

**
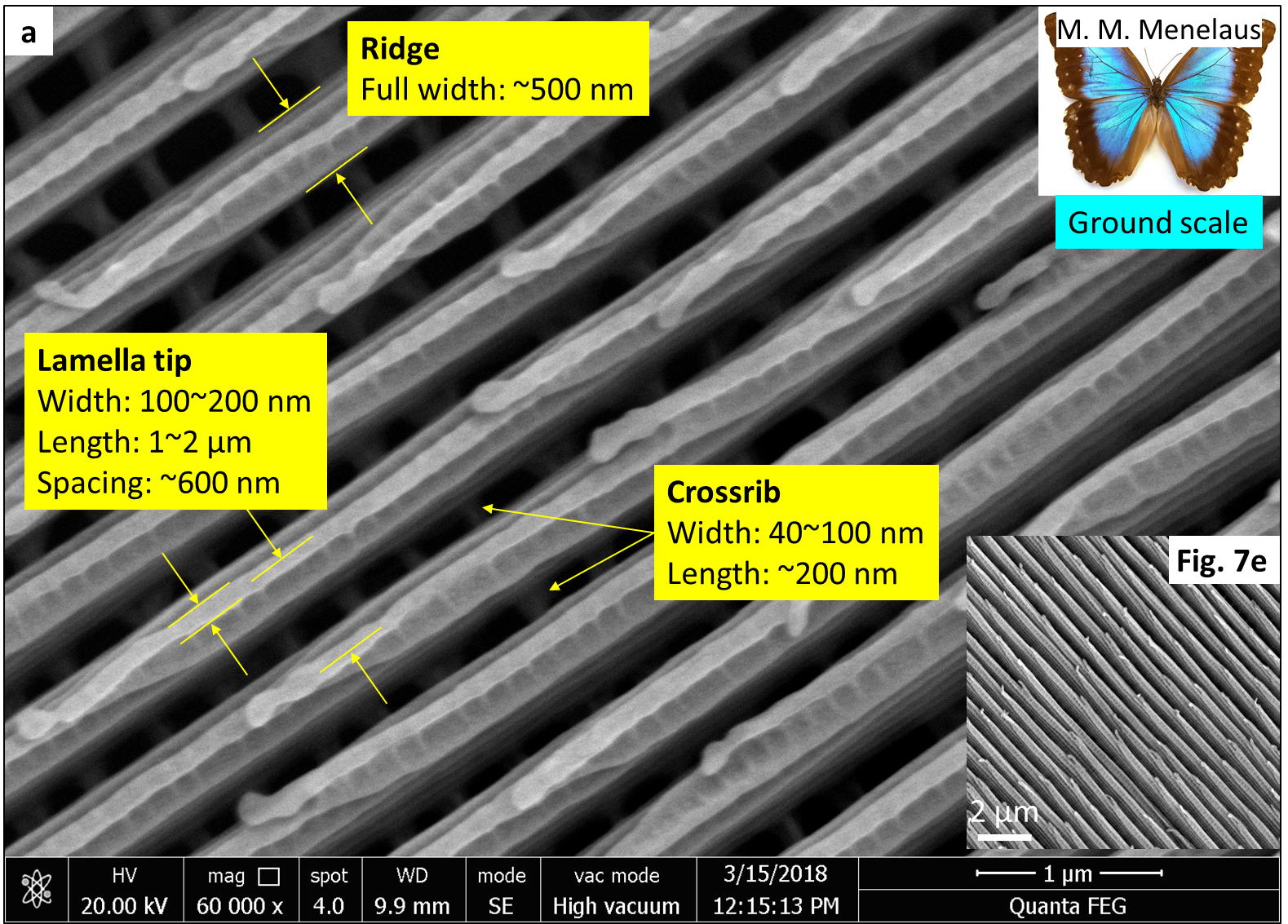
**

**
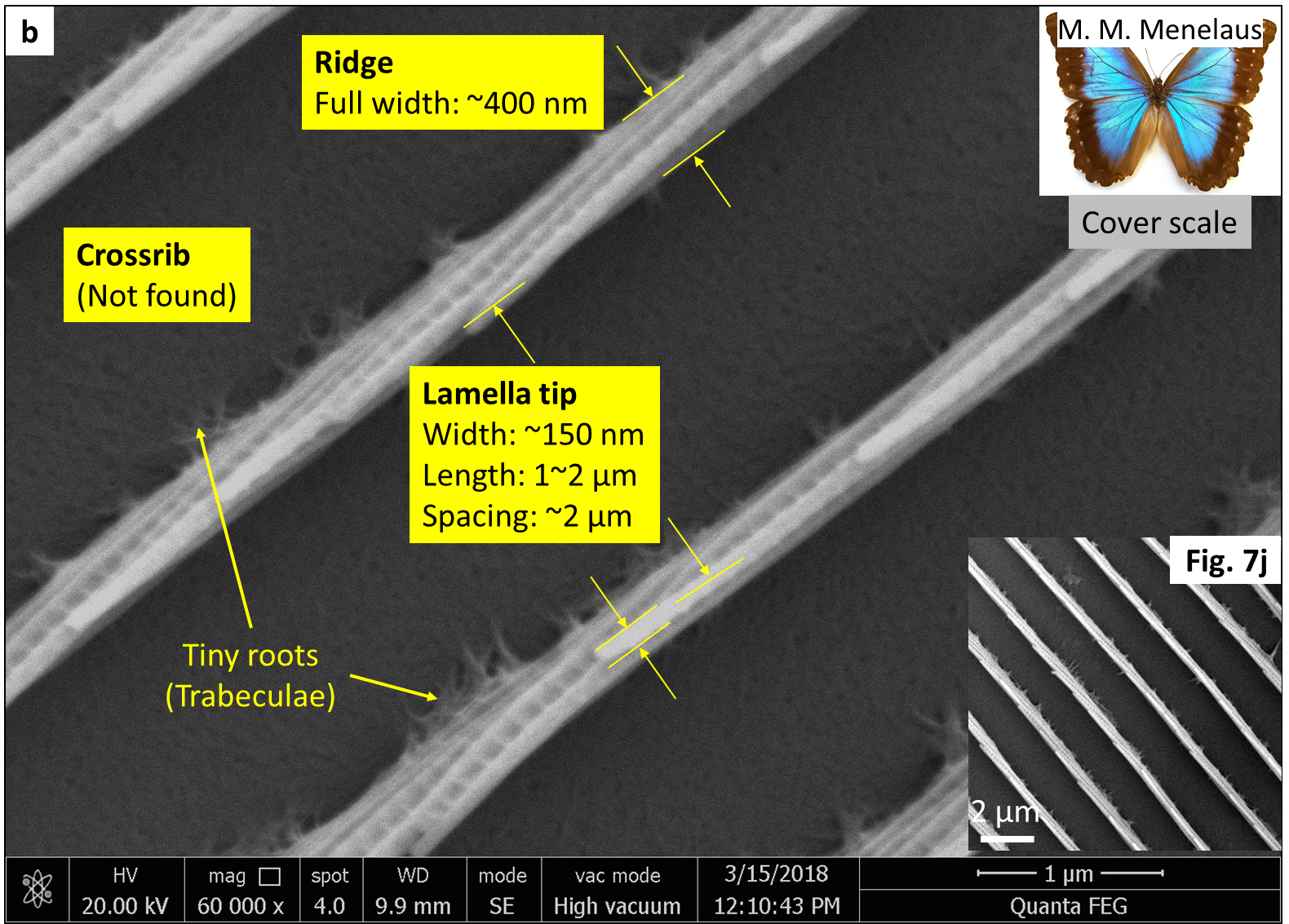
**

**Figure S5. High-resolution SEM images of M. m. menelaus wing scales.** **a** Ground scale. **b** Cover scale. Features of Lamella tip, Ridge and Crossrib are marked with scales.

**
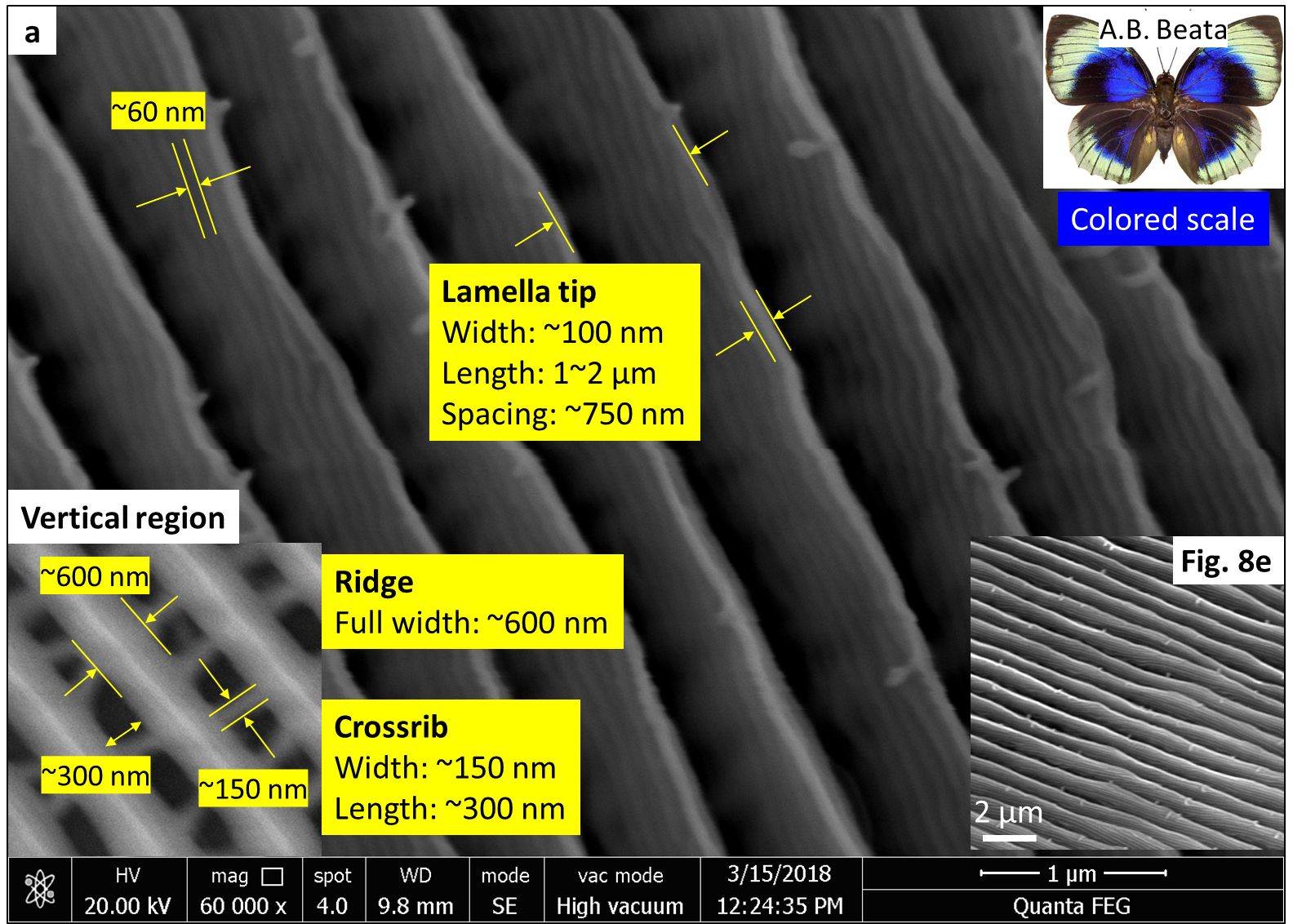
**

**
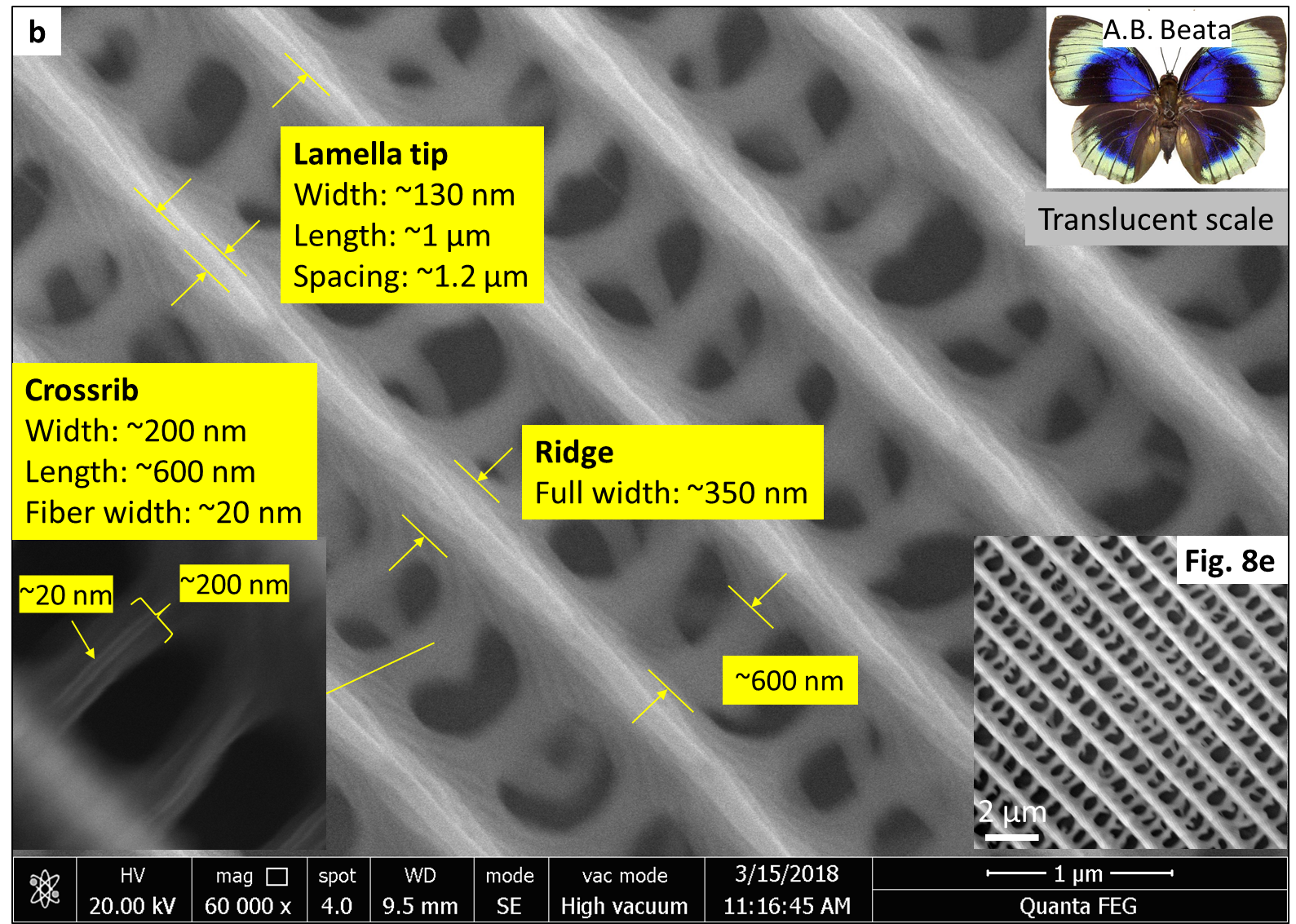
**

**Figure S6. High-resolution SEM images of A. b. beata wing scales.** **a** Colored scale. **b** Translucent scale. Features of Lamella tip, Ridge and Crossrib are marked with scales.


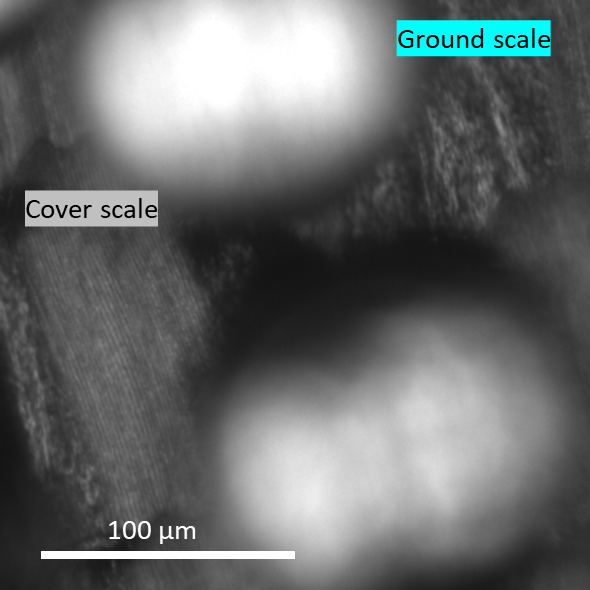


**Figure S7. Optical image of M. m. menelaus wing scales taken by 10x (NA 0.30) objective.** The ridges having ~2-µm spacing can be identified from the cover scale as marked by the yellow arrows.

**Table-S1.** The magnification factors and parameters used in the bandpass filter treatment for Fig. 5.

| **Images:** | **k** | **l** | **m** | **n** | **O** |
| --- | --- | --- | --- | --- | --- |
| Magnification factor | 6.10x | 6.86x | 1.34x | 1x (Ref) | 4.34x |
| Bandpass filter applied [px] | 3 to 14 | 3 to 16 | 1 to 3 | 0 to 2 | 2 to 10 (Ref) |
| Target feature size: 27 to 135 nm | | | | | |

**Table-S2.** The magnification factors and parameters used in the bandpass filter treatment for Fig. 8.

| **Images:** | **a** | **b** | **c** | **d** |  |
| --- | --- | --- | --- | --- | --- |
| Magnification factor | 6.11x | 1.53x | 1x (Ref) | 13.37x |  |
| Bandpass filter applied [px] | 5 to 21 | 1 to 5 | 1 to 3 | 10 to 45 (Ref) |  |
| Target feature size: 50 to 200 nm | | | | | |

**Table-S3.** Summary of observation from the butterfly wing scales based on high-resolution SEM images in Fig. S5 and S6.

| **(a) Ground Scales** | **SEM** | **OM** | **SL (~2.85x)** |
| --- | --- | --- | --- |
| Lamella tip width | 100 to 200 nm | N | N |
| Lamella tip length | 1 to 5 µm | N | Y |
| Ridge spacing | ~600 nm | Y | Y |
| Ridge full-width | ~500 nm | Y | Y |
| Crossribs width | 40 to 100 nm | N | N |
| Crossribs length | ~ 200 nm | N | N |

| **(b) Cover Scales** | **SEM** | **OM** | **SL (~2.35x)** |
| --- | --- | --- | --- |
| Lamella tip width | ~150 nm | N | N |
| Lamella tip length | 1 to 2 µm | Y | Y |
| Ridge spacing | ~2 µm | Y | Y |
| Ridge full-width | ~400 nm | Y | Y |
| No crossribs;  only tiny roots | ~40 nm wide; 100 to 400 nm long | N | Y |

| **(c) Colored Scales** | **SEM** | **OM** | **SL (~2.73x)** |
| --- | --- | --- | --- |
| Lamella tip width | ~100 nm | N | N |
| Lamella tip length | 1 to 2 µm | N | N |
| Ridge spacing | ~750 nm | Y | Y |
| Ridge full-width | ~600 nm | Y | Y |
| Crossribs width | ~150 nm | N | N |
| Crossribs length | ~300 nm | N | N |

| **(d) Translucent Scales** | **SEM** | **OM** | **SL (~2.17x)** |
| --- | --- | --- | --- |
| Lamella tip width | ~130 nm | N | N |
| Lamella tip length | ~1 µm | Y | Y |
| Ridge spacing | ~1.2 µm | Y | Y |
| Ridge full-width | ~350 nm | Y | Y |
| Crossribs form networks | ~200 nm wide;  ~600 nm long; contain 20-nm fibers | N | Y |

OM: Optical microscope

SL: Superlens (with estimated magnification factor)

N: Not observable

Y: Observable

Diffraction limited of the optical system: 288 nm
